# Nitric Oxide-Releasing *S*-Nitrosoglutathione-Conjugated Poly(Lactic-*Co*-Glycolic Acid) Nanoparticles for the Treatment of MRSA-Infected Cutaneous Wounds

**DOI:** 10.3390/pharmaceutics12070618

**Published:** 2020-07-02

**Authors:** Juho Lee, Dongmin Kwak, Hyunwoo Kim, Jihyun Kim, Shwe Phyu Hlaing, Nurhasni Hasan, Jiafu Cao, Jin-Wook Yoo

**Affiliations:** 1 College of Pharmacy, Pusan National University, Busan 46241, Korea; jhlee2350@gmail.com (J.L.); kdm318@naver.com (D.K.); rlagusdn0628@naver.com (H.K.); jihyunjoshkim@gmail.com (J.K.); shwephyuhlaing@gmail.com (S.P.H.); hasni1986.nh@gmail.com (N.H.); caojiafu1985@163.com (J.C.); 2 Department of Cogno-Mechatronics Engineering, College of Nanoscience & Nanotechnology, Pusan National University, Busan 46241, Korea

**Keywords:** *S*-nitrosoglutathione (GSNO), poly(lactic-*co*-glycolic acid) (PLGA), nitric oxide, nitric oxide-releasing nanoparticles, GSNO-conjugated PLGA, methicillin-resistant *Staphylococcus aureus* (MRSA), infected wound healing

## Abstract

*S*-nitrosoglutathione (GSNO) has emerged as a potent agent for the treatment of infected cutaneous wounds. However, fabrication of GSNO-containing nanoparticles has been challenging due to its high hydrophilicity and degradability. The present study aimed to fabricate nanoparticles using newly synthesized GSNO-conjugated poly(lactic-*co*-glycolic acid) (PLGA) (GSNO-PLGA; GPNPs). Since hydrophilic GSNO was covalently bound to hydrophobic PLGA, loss of GSNO during the nanoparticle fabrication process was minimized, resulting in sufficient loading efficiency (2.32% of GSNO, 0.07 μmol/mg of NO). Real-time NO release analysis revealed biphasic NO release by GPNPs, including initial burst release within 3 min and continuous controlled release for up to 11.27 h, due to the differential degradation rates of the –SNO groups located at the surface and inside of GPNPs. Since GPNPs could deliver NO more efficiently than GSNO in response to increased interaction with bacteria, the former showed enhanced antibacterial effects against methicillin-resistant *Staphylococcus aureus* (MRSA) at the same equivalent concentrations of NO. Finally, the facilitating effects of GPNPs on infected wound healing were demonstrated in MRSA-challenged full-thickness wound mouse model. Collectively, the results suggested GPNPs as an ideal nanoparticle formulation for the treatment of MRSA-infected cutaneous wounds.

## 1. Introduction

Cutaneous wound infection is an intractable global problem that costs millions of dollars per year for treatment [1,2]. Moreover, it can cause life-threatening complications, including sepsis, whose mortality is approximately 30% in the United States [3]. In general, uninfected cutaneous wounds can heal spontaneously via the three sequential wound healing processes: inflammation, cell proliferation, and tissue remodeling [4,5]. However, wounds can be frequently contaminated by bacteria that induce chronic inflammation, resulting in impaired wound healing [6,7]. Therefore, the eradication of infected bacteria from the wound site is the primary step to facilitate wound healing [8].

For the treatment of infected wounds, topical antibiotics are widely used due to their potent effects on decreasing microbial burden at the wound site [9]. However, due to overuse of a wide range of antibiotics, development of drug-resistant bacteria, including methicillin-resistant *Staphylococcus aureus* (MRSA), has emerged as a serious problem; more than two million people are infected and approximately 700,000 patients die as a result every year [10,11,12]. Although a few antibiotics, such as vancomycin, are currently used for the treatment of MRSA infection, emergence of vancomycin-resistant strains, namely vancomycin-resistant enterococci (VRE) and vancomycin-resistant *Staphylococcus aureus* (VRSA) has been reported, [13]. Therefore, the development of novel therapeutics, which are functional against drug-resistant bacteria without concerns of inducing resistance, is imperative for the treatment of infected cutaneous wounds [14].

In recent decades, nitric oxide (NO) has gained attention as a novel agent for healing infected wounds due to its beneficial effects in modulating inflammation and facilitating wound healing processes, such as cell proliferation and tissue remodeling [15,16,17,18,19]. Besides its wound-healing promoting effects, NO possesses broad-spectrum antibacterial activity, which could be a desirable property for the treatment of infected wounds [20]. By forming reactive nitrogen species or by direct nitrosation, NO can damage bacterial cell membranes, proteins, and DNA, resulting in bacterial cell death [21,22]. Due to multiple antibacterial mechanisms, NO can exert antibacterial effects on drug-resistant bacterial strains, including MRSA [23]. In addition, developing resistance against NO and surviving would require the accumulation of multiple favorable mutations over several generations, which seems quite remote [24]. Taken together, NO-based formulations seem to be desirable therapeutics for the treatment of infected cutaneous wounds. Moreover, with advancements in nanotechnology that have been utilized for enhanced therapeutic effects [25,26], an array of NO-releasing nanoparticle formulations have been recently developed and evaluated, including polyethyleneimine-NONOates-doped PLGA nanoparticles, NO-releasing silica nanoparticles, and NO-releasing metal nanoparticles, all of which have shown enhanced antibacterial and wound healing activities [20,27,28,29,30,31]. However, these NO-releasing nanoparticle formulations achieved limited success since most of them consisted of non-biodegradable materials, such as polyethyleneimine and heavy metals, that can potentially accumulate in the skin tissue [32,33,34]. To avoid potential toxicity, the development of NO-releasing nanoparticles consisting of biocompatible materials is highly desirable.

S-nitrosoglutathione (GSNO) is an endogenously synthesized NO donor that changes to glutathione (GSH) after releasing NO, the non-toxic and most abundant antioxidant in mammalian cells [35,36]. Due to its endogeneity, various GSNO formulations, such as hydrogels, films, and microparticles, have been developed as wound dressings [37,38,39,40]. However, until now, the fabrication of a nanoparticle formulation using GSNO has been challenging owing to the high hydrophilicity of GSNO, which remains a big hurdle to achieve sufficient loading of NO. Although several GSNO-loaded nanoparticles have been developed to date [41,42,43,44], their in vivo therapeutic efficacy has not yet been reported, possibly due to insufficient GSNO loading.

In this study, to generate a sufficient amount of GSNO-incorporated nanoparticles, we synthesized GSNO-conjugated poly(lactic-co-glycolic acid) (PLGA) (GSNO-PLGA), and fabricated nanoparticles using the novel polymer for the treatment of a MRSA-infected wound. By conjugating a small hydrophilic GSNO molecule with hydrophobic PLGA, the loss of GSNO during the nanoparticle fabrication process could be minimized, resulting in increased NO loading. Further, GSNO-PLGA and GSNO-PLGA nanoparticles (GPNPs) were characterized in a series of in vitro experiments. After characterization of the physicochemical properties of GPNPs, the enhanced antibacterial effects towards MRSA were examined. Moreover, for exploring GSNO nanoparticle formulation as a drug for infected wound healing, the in vivo wound healing effect of GPNPs was evaluated for the first time using a MRSA-challenged full-thickness wound mouse model.

## 2. Materials and Methods

### 2.1. Materials

Poly(d,l-lactide-*co*-glycolide) (PLGA, lactide/glycolide = 50/50, molecular weight (MW): 1728 Da, acid end cap) was purchased from PolySciTech (AP037, Akina, Inc., West Lafayette, IN, USA). Glutathione (GSH, reduced form), and *N*-hydroxysuccinimide (NHS) were purchased from Wako Pure Chemical (Osaka, Japan). Poly vinyl alcohol (PVA), Mayer’s hematoxylin, tert-amyl alcohol, 2,2,2-tribromoethanol, and sodium nitrite were purchased from Sigma-Aldrich (St. Louis, MO, USA). Bacto™ tryptic soy broth (TSB) was purchased from BD Biosciences (San Jose, CA, USA). Agar, *N*-(3-dimethylaminopropyl)-*N*’-ethylcarbodiimide hydrochloride (EDC), and eosin-Y solution were purchased from Daejung Chemicals & Metals (Siheung, Korea). A Twort’s Gram stain set was purchased from Newcomer Supply (Middleton, WI, USA). Masson’s trichrome stain kit was purchased from Abcam (Cambridge, MA, USA). The LIVE/DEAD^®^ BacLight™ bacterial viability kit was procured from Thermo Fisher Scientific (Waltham, MA, USA). All other reagents and solvents were of the highest analytical grade commercially available.

### 2.2. Synthesis of GSNO

GSNO was synthesized by the method previously described, with some modifications [45]. Briefly, GSH and sodium nitrite were dissolved in cold HCl solution (final concentrations of sodium nitrite, GSH, and HCl were adjusted to 0.625 M) and stirred at 300 rpm for 20 min. GSNO was separated from the solution by adding excessive amount of acetone, and the precipitate was washed twice with acetone and thrice with ether. Residual solvent was removed by vacuum drying and GSNO was stored at −20 °C till future use.

### 2.3. Synthesis of GSNO-Conjugated PLGA

GSNO-conjugated PLGA (GSNO-PLGA) was synthesized via an EDC/NHS coupling reaction between the carboxylic group of PLGA and the primary amine group of GSNO. First, PLGA was activated using EDC/NHS as previously described with some modifications [46]. Briefly, for PLGA activation, 5 g of PLGA, 1.13 g of EDC, and 0.67 g of NHS (molar ratio of PLGA:EDC:NHS = 1:2:2) were dissolved in 30 mL of DMSO and incubated for 24 h at room temperature. PLGA-NHS was collected and purified by adding an excessive amount of distilled water (DW), and eventually freeze-dried to remove residual DW. Dried PLGA-NHS was stored at −20 °C. For conjugation of GSNO to PLGA, 4 g of PLGA-NHS and 1.6 g of GSNO (molar ratio of PLGA-NHS:GSNO = 1:2) were dissolved in 30 mL of DMSO and incubated for 24 h at room temperature. After incubation, GSNO-PLGA was collected by adding an excessive amount of DW, followed by centrifugation at 20,000× g for 10 min. The pellets were collected and washed twice with ice-cold DW to remove unconjugated GSNO. Subsequently, GSNO-PLGA was freeze-dried and stored at −20 °C.

Synthesis of GSNO-PLGA was confirmed by proton nuclear magnetic resonance (^1^H-NMR) spectroscopy (Avance NEO 500 MHz, Bruker, Germany). Samples were dissolved in DMSO-d6 (Alfa Aesar, Ward Hill, MA, USA) before ^1^H-NMR analysis. The presence of -SNO groups in GSNO-PLGA was analyzed using UV/Vis spectroscopy. UV/Vis spectra were recorded using different concentrations of GSNO-PLGA in DMSO.

### 2.4. Fabrication of GPNPs

GSNO-PLGA NPs (GPNPs) were fabricated using an oil-in-water emulsion solvent evaporation method. Briefly, 100 mg of GSNO-PLGA was dissolved in 4 mL of dichloromethane (DCM) and poured into 20 mL of ice-cold 1% PVA solution. The mixture was sonicated at 150 W for 90 s and stirred at 550 rpm for 4 h in ice slurry to remove DCM by evaporation. After solvent evaporation, GPNPs were collected, washed twice by centrifugation (20,000× *g*, 30 min), and freeze-dried for future use (50 mg of trehalose was added for cryoprotection).

After fabrication of GPNPs, scanning electron microscopy (SEM, SUPRA 25, Carl Zeiss, Jena, Germany) was performed to investigate their morphology. Hydrodynamic particle size and the zeta potential of GPNPs were measured using a Zetasizer Nano ZS90 (Malvern Instruments, Worcestershire, UK) [47,48]. The loading efficiency of GPNPs was measured using a Sievers Nitric Oxide Analyzer (NOA 280i, GE Analytical Instruments, Boulder, CO, USA). The instrument was calibrated with NO-free air and a 45-ppm NO gas standard. NO gas released from the medium was transported to the instrument at a flow rate of 80 mL/min by Ar gas. After instrument stabilization, 5 mg of GPNPs were dissolved in 1 mL of 1 M NaOH solution and added to the fast-degrading medium at 37 °C, consisting of phosphate buffered saline (PBS) and 30 mM ascorbic acid. GSNO loading efficiency was calculated as per Equation (1).

GSNO loading (%) = NO loading (μmol/mg)/1000 × 336.32 × 100(1)

### 2.5. NO Release from GPNPs

The real-time NO release profile of GPNPs was analyzed using a NOA 280i by the same method described above, except for the medium. Simulated wound fluid (SWF), composed of 0.64% NaCl, 0.22% KCl, 2.5% NaHCO_3_, and 0.35% NaH_2_PO_4_ with pH adjusted to 7.4, was used as a releasing medium. After instrument stabilization, 50 mg of GPNPs were introduced into the medium under light protection.

### 2.6. Antibacterial Assay

The enhanced antibacterial effects of GPNPs were investigated relative to those of GSNO. The colony forming unit (CFU) method was used for quantitative evaluation of the antibacterial effect against MRSA (USA300). Briefly, MRSA was incubated in tryptic soy broth (TSB) at 37 °C with shaking (200 rpm) for 24 h before initiating the experiment. Thereafter, MRSA was washed twice with PBS and diluted until the optical density at 600 nm reached 0.15 to 0.2. After adjusting the concentrations of the MRSA suspension, 0.5, 1, and 2 mM equivalent concentrations of NO in GPNPs and GSNO were added and incubated at 37 °C for 24 h. The samples were serially diluted and 100 μL of each dilution was plated on TSB-agar plates. After overnight incubation at 37 °C, colonies were counted and bacterial concentration was calculated.

For visualizing antibacterial effects, an MRSA suspension treated with 2 mM NO-containing GPNPs and GSNO at 37 °C for 24 h was stained with LIVE/DEAD^®^ BacLight™ bacterial viability kit consisting of SYTO 9 (Thermo Fisher Scientific, Waltham, MA, USA) and propidium iodide (PI), according to the manufacturer’s protocol. After staining, samples were immediately visualized using a fluorescence confocal laser scanning microscope (LSM 800, Carl Zeiss, Oberkochen, Germany) at a magnification of 20×.

### 2.7. In vivo Wound Healing Study in an MRSA-Challenged Full-Thickness Wound Mouse Model

#### 2.7.1. Macroscopic Assessment: Wound Size Reduction

All animal experiments were approved by Pusan National University Institutional Animal Care and Use Committee (PNU-IACUC) (approval date: 17 April 2020; approval number: PNU-2020-2590). Six-week-old male imprinted control region (ICR) mice were purchased from Samtako Bio Korea (Osan, Korea) and acclimatized for a week before experiment initiation. For full-thickness wound generation, mice were anesthetized with 0.6 mg/g of avertin (2,2,2-tribromoethanol) via intraperitoneal injection. After anesthesia, hair on the dorsal area was removed using electric trimmers and hair removal cream (Veet (for sensitive skin), Reckitt Benckiser, Slough, UK). A full-thickness wound was created on the dorsal area of mice using an 8-mm diameter disposable biopsy punch (Kai medical, Tokyo, Japan). To induce MRSA infection, 6 × 10^5^ CFU of MRSA were introduced into each wound, and the latter was covered with Tegaderm film and Durapore surgical tape (3M, St. Paul, MN, USA) as a protective dressing. After 2 days, mice were divided into three groups: GPNP treatment group, GSNO treatment group, and untreated group. Photographs of the wounds were taken before drug treatment, and 20 mg of GPNPs and the same equivalent amount of NO in GSNO were applied to each wound before covering them with a protective dressing, which was changed every alternate day. The wound size was measured using ImageJ software.

#### 2.7.2. Histological Assessment

On day 8, all mice were sacrificed, and wound tissues were collected for histological assessment using an 8-mm biopsy punch. Samples were fixed in 10% buffered formalin solution for 24 h and embedded in paraffin blocks. Five-micron sections were obtained, using microtome (Shandon, Pittsburgh, PA, USA), for staining. Hematoxylin and eosin (H&E) staining, Masson’s trichrome staining, and Twort’s Gram staining were performed according to the manufacturer’s protocol.

### 2.8. Statistics

All statistical analyses were conducted using a one-way analysis of variance (ANOVA) or two-way ANOVA, followed by Bonferroni’s post-hoc test equipped with GraphPad Prism 5.0 (GraphPad Software, Inc., La Jolla, CA, USA). Results with p-values less than 0.05 were considered statistically significant.

## 3. Results

### 3.1. Synthesis of GSNO-Conjugated PLGA

**Figure 1:**
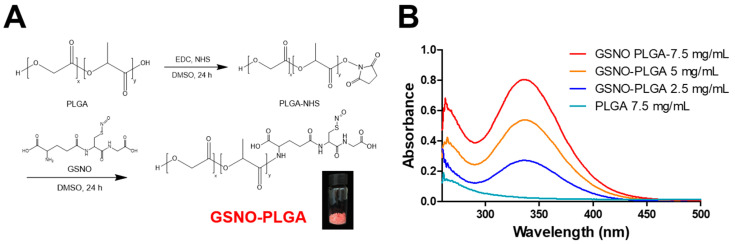

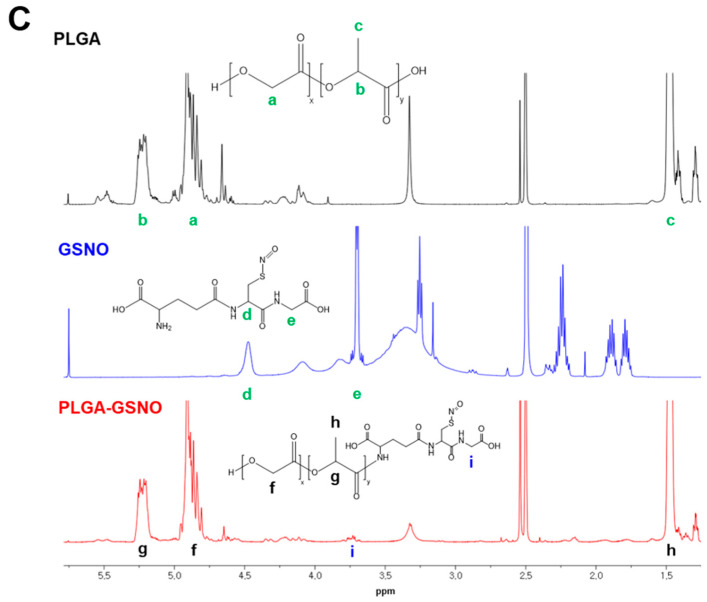
Synthesis of S-nitrosoglutathione-conjugated poly(lactic-co-glycolic acid) (GSNO-PLGA). (**A**) Synthesis scheme of GSNO-PLGA. (**B**) UV/Vis spectra of various concentrations of GSNO-PLGA dissolved in DMSO. (**C**) ^1^H-NMR spectrum of PLGA, GSNO, and GSNO-PLGA in DMSO-d6. EDC: *N*-(3-dimethylaminopropyl)-*N*’-ethylcarbodiimide hydrochloride; NHS: *N*-hydroxysuccinimide.

GSNO-PLGA was synthesized via an EDC/NHS coupling reaction between the carboxylic group of PLGA and the primary amine group of GSNO (Figure 1). After synthesis and purification, hydrophobic pink-colored GSNO-PLGA was successfully collected. To confirm the presence of the *S*-nitrosothiol group in GSNO-PLGA, UV/Vis spectroscopy was conducted. As shown in Figure 1, a concentration-dependent characteristic peak was observed at a wavelength of approximately 335 nm, which matched with the previously reported peak of -SNO groups in GSNO [35]. In the ^1^H-NMR spectra of GSNO-PLGA, big peaks from PLGA and small peak from GSNO were observed (Figure 1C). Since one GSNO molecule was conjugated to PLGA, which possesses more than 10 repeating units of glycolic and lactic acids, the peak area of GSNO was much smaller than that of PLGA. In addition, physicochemical properties, such as solubility, resembled those of PLGA more than those of GSNO, since the majority of GSNO-PLGA was made of the PLGA moiety.

### 3.2. Fabrication of GPNPs

**Figure 2:**
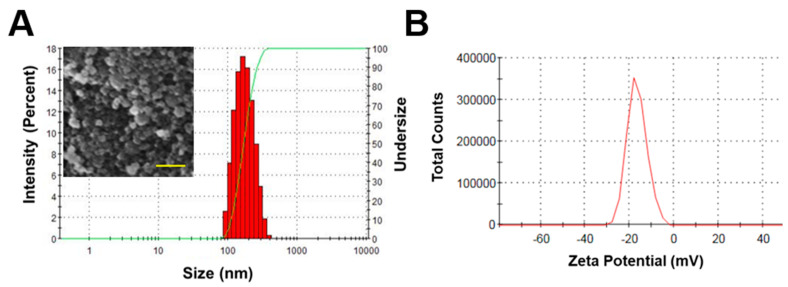
Characterization of GPNPs. (**A**) Scanning electron microscopy (SEM) images and particle size distribution of GPNPs. Scale bar = 0.5 μm. (**B**) Zeta potential distribution of GPNPs at pH 7.4.

After synthesizing GSNO-PLGA, GPNPs were fabricated using the oil-in-water emulsion solvent evaporation method. As shown in Figure 2A, globular-shaped GPNPs were observed. In addition, dynamic light scattering (DLS) measurement revealed the hydrodynamic diameter of GPNPs was 164.5 ± 2.2 nm, with a narrow poly disperse index (PDI) (0.12 ± 0.03) (Table 1, Figure 2A). Surface charge characteristics of GPNPs were analyzed using a Zetasizer, and the mean zeta potential value was −17 ± 0.6 mV (Table 1, Figure 2B). Owing to the presence of carboxylic groups in GSNO, GPNPs showed a negative zeta potential. The amount of NO incorporated into GPNPs was confirmed using a NOA. GPNPs were dissolved in 1 M NaOH solution and introduced into the degrading medium to facilitate NO liberation from the nanoparticles. Approximately 0.07 ± 0.01 μmol/mg of NO was loaded into GPNPs, which corresponded to 14.3% of theoretically ideal NO loading. Owing to the easily degradable nature of NO donors, including GSNO, it was practically the highest loading in GPNPs.

### 3.3. NO Release from GPNPs

**Figure 3:**
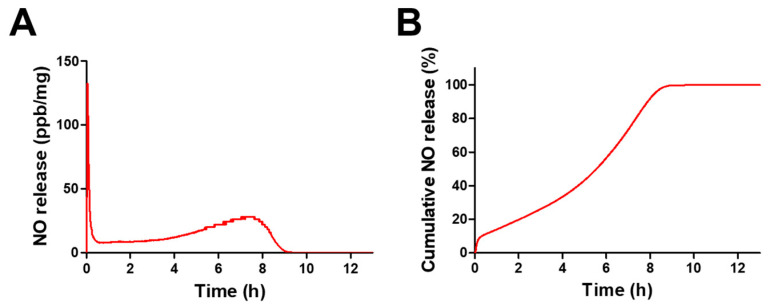
NO release profile of GPNPs in simulated wound fluid (SWF) at 37 °C. (**A**) Real-time NO release profile of GPNPs. (**B**) Cumulative NO release profile of GPNPs.

The release profile of GPNPs was evaluated in SWF at 37 °C using a NOA. As shown in Table 2 and Figure 3, GPNPs released NO for more than 11 h in a biphasic release pattern. Burst release of NO was observed within 3 min of experiment initiation, due to the fast decomposition of –SNO groups located on the surface of GPNPs (Figure 3A). After initial burst release, GPNPs continuously released NO for 8 h in a controlled manner, and residual NO in GPNPs was released up to 11.27 h. Overall, 10% of NO was immediately released within 3 min, 90% was constantly released for 8 h, and less than 1% was released for 4 h (Figure 3B).

### 3.4. Antibacterial Assay

**Figure 4:**
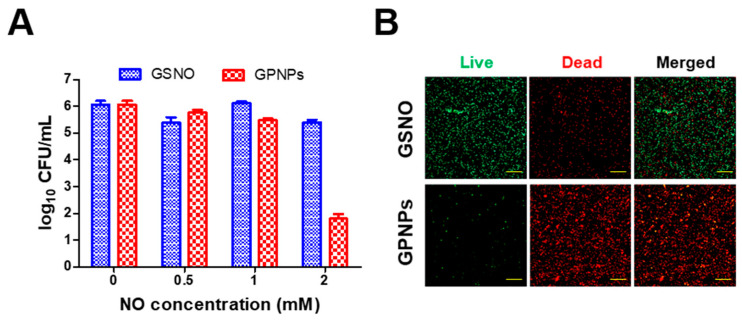
Antibacterial effects of GPNPs against methicillin-resistant *Staphylococcus aureus* (MRSA) compared to that of GSNO. (**A**) Enhanced antibacterial effects of GPNPs by the colony forming unit (CFU) method. MRSA was incubated with GPNPs and GSNO at 37 °C for 24 h. Data are presented as the mean ± SD (*n* = 3). (**B**) Fluorescent confocal microscopic images of MRSA treated with GPNPs and GSNO at 37 °C for 24 h followed by LIVE/DEAD^®^ BacLight™ staining. Green fluorescence (SYTO 9) and red fluorescence (propidium iodide) indicate live and dead bacteria, respectively. Scale bar = 50 μm.

The enhanced antibacterial effects of GPNPs against MRSA were examined by the CFU method and fluorescence confocal microscopy using the LIVE/DEAD^®^ BacLight™ bacterial viability kit. Since the antibacterial effects of GSNO against MRSA have been widely explored in previous studies, in the present study, the antibacterial effects of GPNPs were evaluated in comparison to those of GSNO. As shown in Figure 4A, GPNPs showed antibacterial effects at a concentration of 2 mM NO, whereas GSNO did not exhibit any such effect at the same equivalent concentration of NO. More than a four-log reduction of MRSA was observed in the GPNPs-treated groups incubated with 2 mM NO equivalent of GPNPs for 24 h at 37 °C. Due to their increased interaction with MRSA, GPNPs showed an enhanced antibacterial effect against MRSA than GSNO.

The increased antibacterial effects of GPNPs were visualized using the LIVE/DEAD^®^ BacLight™ bacterial viability kit, containing a widely used fluorescence dye consisting of PI and SYTO 9 for distinguishing normal bacterial cells from damaged cells. As shown in Figure 4B, distinct red fluorescence was observed in the images from the GPNPs-treated group, while prominent green fluorescence was observed in the GSNO-treated group at the same equivalent concentrations of NO (2 mM). Since PI cannot penetrate normal bacterial cells, red fluorescence signals were considered to represent damaged bacteria while green fluorescence from SYTO 9, which can penetrate all bacterial cell membranes, was considered to represent normal bacteria. Owing to the enhanced antibacterial effects, the majority of the MRSA incubated with GPNPs for 24 h were dead, whereas those from the GSNO-treated group survived due to insufficient antibacterial activity.

### 3.5. In vivo Wound Healing Study

#### 3.5.1. Macroscopic Assessment: Wound Size Reduction

**Figure 5:**
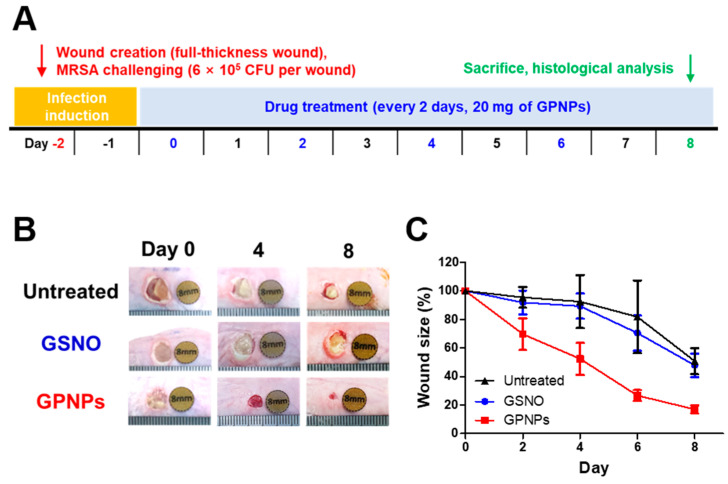
In vivo wound-healing promotion effect of GPNPs in a MRSA-challenged full-thickness wound mouse model. (**A**) Experimental flow. (**B**) Representative macroscopic images of the wound treated with or without GSNO and GPNPs. (**C**) Wound size reduction profiles after treatment initiation (*n* = 5).

Enhanced wound-healing promotion effects of GPNPs were evaluated in a MRSA-challenged full-thickness wound mouse model. Two days after wound creation and MRSA challenge (day 0), a yellowish biofilm formed at the wound site due to replicating MRSA. As shown in Figure 5, the GPNPs-treated group showed reduced wound size relative to the untreated and GSNO-treated groups. Four days after treatment initiation, the wound site of GPNPs-treated mice showed cleaner morphologies than other groups, where biofilms were still visible on the wound site of the GSNO-treated and untreated groups (Figure 5B). On day 8, mean wound size had considerably decreased and was slightly more than 20% of the initial size, whereas that of the GSNO-treated and untreated groups was approximately 50% (Figure 5C). As a result of successful bacterial eradication, GPNPs showed enhanced wound healing effects immediately after treatment initiation. However, GSNO could not facilitate wound healing as effectively, owing to its quick clearance from the wound site and poor biofilm penetration, resulting in decreased bactericidal effects on MRSA.

#### 3.5.2. Histological Assessment

**Figure 6:**
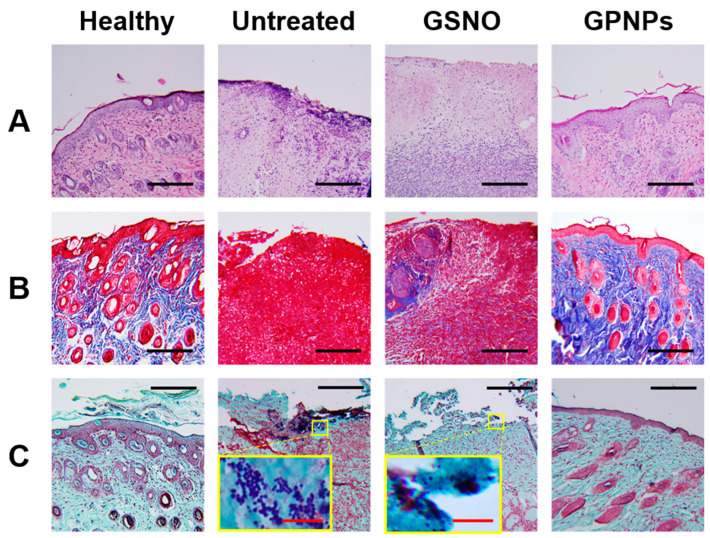
Histological assessment of the wound treated with or without GPNPs and GSNO, on day 8. (**A**) Hematoxylin and eosin (H&E) staining; (**B**) Masson’s trichrome staining (blue color represents collagen); (**C**) Twort’s Gram staining. Dark blue colored spots represent Gram-positive bacteria (MRSA). Black and red scale bars = 200 μm and 10 μm, respectively.

For further investigation of wound healing, wound tissues were paraffin-embedded, and morphologies were analyzed by H&E staining, Masson’s trichrome staining, and Twort’s Gram staining. As shown in Figure 6A, the GPNPs-treated group but not the GSNO-treated and untreated groups exhibited morphologies similar to the healthy group. Distinct epidermis and hair follicles were also observed in H&E-stained images of healthy and GPNPs-treated groups, while large numbers of immune cells and granulation tissue were observed in the GSNO-treated and untreated groups. To evaluate collagen deposition in the dermis of the wounds, Masson’s trichrome staining was performed. Images from the healthy and GPNPs-treated groups showed prominent blue-colored regions in dermis, which indicated the presence of a large amount of collagen synthesized by fibroblasts (Figure 6B). Conversely, since the majority of the dermis in tissues from GSNO-treated and untreated groups consisted of granulocytes and immune cells that are unable to produce collagen matrix, only a small blue colored region was observed. To visualize bacteria at the wound site, Twort’s Gram staining was conducted. In Figure 6C, a large number of round-shaped, 1- to 2-μm-sized purple dots representing Gram-positive cocci (MRSA) were observed in GSNO-treated and untreated groups. In contrast, in healthy and GPNPs-treated groups, the purple dots were rarely seen, implying low levels of bacteria that were hard to detect by Gram staining [39,49]. Collectively, the GPNPs-treated group showed recovered morphology, with increased collagen deposition and tissue remodeling compared to the GSNO-treated and untreated groups as a result of enhanced bacterial removal from the wound site.

## 4. Discussion

Although NO-releasing therapeutics using GSNO have gained attention due to their biocompatibility (endogenously synthesized from glutathione), the fabrication of a nanoparticle formulation of GSNO retaining the benefits of nanoparticles as well as GSNO has been challenging due to the highly hydrophilic nature of GSNO which limits loading efficiency and reduces therapeutic efficacy. In this study, GPNPs were fabricated using a newly synthesized GSNO-PLGA to achieve sufficient loading of GSNO.

GSNO-PLGA was synthesized by an EDC/NHS coupling reaction between the carboxylic group of PLGA and the primary amine group of GSNO (Figure 1A). Since GSNO-PLGA mainly consisted of PLGA (molecular weight ratio of GSNO:PLGA = 1:5), peaks from the GSNO moiety were not as clearly detectable by ^1^H-NMR spectroscopy as those from the PLGA moiety, which has a large number of repeating groups of lactic and glycolic acids (Figure 1C). In addition, the presence of the NO-releasing moiety in GSNO (–SNO group) was confirmed using UV/Vis spectroscopy with the detection of characteristic peaks from the -SNO group at approximately 335 nm (Figure 1B).

After characterization of GSNO-PLGA, GPNPs were fabricated using the oil-in-water emulsion evaporation method. As shown in Figure 2, SEM and hydrodynamic size measurement revealed GPNPs to be of globular shape, with a mean particle size of 164.5 ± 2.2 nm. There were no significant changes in the mean particle size after 24 h, indicating that GPNPs can be stable during application to the wounds (data not shown). Owing to the presence of carboxylic groups in GSNO-PLGA, the surface of GPNPs was negatively charged (zeta potential: −17 ± 0.6 mV). Since the GSNO molecule was conjugated to the hydrophobic PLGA, loss of GSNO was prevented during the nanoparticle fabrication processes, resulting in sufficient loading efficiency of NO (2.32 ± 0.27% of GSNO, 0.07 ± 0.01 μmol/mg of NO).

To investigate NO-release pattern of GPNPs, real-time NO-release analysis was performed using a NOA 280i. Interestingly, GPNPs showed biphasic NO release in SWF at 37 °C (Figure 3). Since -SNO groups in GSNO-PLGA (located on the surface of GPNPs) were exposed to the medium, burst release of NO was observed within 3 min of introducing GPNPs to SWF. On the contrary, wetting of –SNO groups located inside the GPNPs was suppressed by the hydrophobic PLGA moieties of GSNO-PLGA; therefore, hydrolysis of –SNO occurred in a sustained manner, resulting in the controlled release of NO from GPNPs for up to 11.27 h. This may have resulted from the degradation and erosion of the PLGA moiety that caused multiphasic drug release from the PLGA particles [50,51,52].

The antibacterial effects of GPNPs against MRSA were examined by comparing with the effects of GSNO (Figure 4). Since nanoparticle formulations were reported to show enhanced antibacterial effects over their molecular form via increased interaction with 1- to 2-μm-sized bacteria [20,53,54,55], GPNPs were considered to deliver NO to bacteria more efficiently than GSNO. Furthermore, owing to the short migration distance of NO under physiological conditions (40–200 μm, 5 s) [56], the increased delivery efficiency of NO resulted in enhanced antibacterial activity. Therefore, GPNPs that could release NO after coming in contact with bacteria showed more potent antibacterial activity than GSNO against MRSA.

Based on the above in vitro experiments, we expected GPNPs to facilitate infected wound healing by the rapid eradication of bacteria from the wound site. To prove this hypothesis, an in vivo wound healing study was conducted using an MRSA-challenged full-thickness wound mouse model. After infection induction, although daily treatment would have been better considering the NO release profile, GSNO and GPNPs were applied at each wound site every alternate day to avoid the stress caused by frequent anesthesia while changing dressings. On day 0, all wounds were covered with yellowish biofilms of MRSA. After treatment with GPNPs, the biofilms disappeared and wound size reduced compared to GSNO-treated and untreated groups due to the prompt eradication of MRSA from the wound site (Figure 5). Conversely, GSNO did not show any wound healing promoting effects due to the fast clearance of GSNO from the wound site and low penetration across the MRSA biofilm, which led to decreased bactericidal effects at the wound site. On day 8, all mice were sacrificed and wound tissues were collected for histological analysis. Owing to facilitated wound healing, tissues from the GPNPs-treated group were morphologically more similar to normal tissues than those from the GSNO-treated and untreated groups. Tissues from the GPNPs-treated group were well-differentiated (hair follicles, glands, and clear epidermis were observed) with abundant collagen deposition in the dermis region (Figure 6A,B). In contrast, tissues from the GSNO-treated and untreated groups showed an inflammatory environment characterized by the presence of a large number of immune cells and granulocytes. In addition, MRSA could easily be observed at the outer region of wound tissues taken from GSNO-treated and untreated mice, inhibiting wound healing and causing continuous inflammation, whereas bacteria were rarely observed in GPNPs-treated and healthy mice (Figure 6C). Since inflammation was ongoing in the GSNO-treated and untreated groups, tissue remodeling processes were inhibited, resulting in decreased collagen deposition and compromised recovery of the dermis and epidermis. Taken together, as a result of enhanced antibacterial effects, GPNPs showed potent wound healing promotion effects in an MRSA-challenged full-thickness wound mouse model.

## 5. Conclusions

In this study, GPNPs were fabricated using newly synthesized GSNO-PLGA for the treatment of infected cutaneous wounds. Since small hydrophilic GSNO molecules were covalently conjugated to hydrophobic PLGA, loss of GSNO during nanoparticle fabrication was minimized, resulting in the successful fabrication of sufficiently GSNO-loaded nanoparticles. In real-time NO release analysis, a biphasic release pattern was observed in GPNPs, owing to initial burst-release of NO from surface -SNO groups and continuous release of NO from interior -SNO groups in a sustained manner. GPNPs showed enhanced antibacterial effects against MRSA, relative to GSNO, owing to their increased interaction with MRSA and the delivery efficiency of NO to MRSA. Most importantly, GPNPs exhibited wound-healing promotion effects in an MRSA-challenged full-thickness mouse model. These results suggest that GPNPs can be used as a desirable formulation for the treatment of MRSA-infected cutaneous wounds.

## Figures and Tables

**Table 1:** Physicochemical properties of GPNPs.

Particle Size (Hydrodynamic Diameter) (nm)	Poly-Disperse Index	Zeta-Potential (mV)	Loading (GSNO, %)	Loading (NO, μmol/mg)
164.5 ± 2.2	0.12 ± 0.03	-17 ± 0.6	2.32 ± 0.27	0.07 ± 0.01

Results were expressed as the mean ± standard deviation (SD) (*n* = 3).

**Table 2:** NO release properties of GPNPs.

Formulation	[NO]_T_ (μmol/mg)	[NO]_max_ (ppb/mg)	t[NO]_max_ (h)	t_1/2_ (h)	t_d_ (h)
GPNPs	0.07	132	0.05	5.55	11.27

[NO]_T_: total NO release, [NO]_max_: maximum NO flux, t[NO]_max_: time to reach [NO]_max_, t_1/2_: half-life of NO release, t_d_: duration time (99.9% of NO-released time point).

## References

[1] Daeschlein G. (2013). Antimicrobial and antiseptic strategies in wound management. Int. Wound J..

[2] Lee J., Hlaing S.P., Cao J., Hasan N., Yoo J.-W. (2020). In vitro and in vivo evaluation of a novel nitric oxide-releasing ointment for the treatment of methicillin-resistant Staphylococcus aureus-infected wounds. J Pharm. Investig..

[3] Fleischmann C., Scherag A., Adhikari N.K., Hartog C.S., Tsaganos T., Schlattmann P., Angus D.C., Reinhart K. (2016). Assessment of global incidence and mortality of hospital-treated sepsis. Current estimates and limitations. Am. J. Respir. Crit. Care Med..

[4] Bello Y.M., Phillips T.J. (2000). Recent advances in wound healing. JAMA.

[5] George Broughton I., Janis J.E., Attinger C.E. (2006). The basic science of wound healing. Plast. Reconstr. Surg..

[6] Jones S.G., Edwards R., Thomas D.W. (2004). Inflammation and wound healing: The role of bacteria in the immuno-regulation of wound healing. Int. J. Low. Extrem. Wounds.

[7] Yurt R.W., McManus A.T., Mason A.D., Pruitt B.A. (1984). Increased susceptibility to infection related to extent of burn injury. Arch. Surg..

[8] Choi M., Hasan N., Cao J., Lee J., Hlaing S.P., Yoo J.-W. (2019). Chitosan-based nitric oxide-releasing dressing for anti-biofilm and in vivo healing activities in MRSA biofilm-infected wounds. Int. J. Biol. Macromol..

[9] Dabiri G., Damstetter E., Phillips T. (2016). Choosing a wound dressing based on common wound characteristics. Adv. Wound Care.

[10] Fair R.J., Tor Y. (2014). Antibiotics and bacterial resistance in the 21st century. Perspect. Med. Chem..

[11] Liu S., Cai X., Xue W., Ma D., Zhang W. (2020). Chitosan derivatives co-delivering nitric oxide and methicillin for the effective therapy to the methicillin-resistant S. aureus infection. Carbohydr. Polym..

[12] Behzadi P. (2018). DNA microarrays and multidrug resistant bacteria. Eur. Pharm. Rev..

[13] Heinze K., Kabeto M., Martin E.T., Cassone M., Hicks L., Mody L. (2019). Predictors of methicillin-resistant Staphylococcus aureus and vancomycin-resistant enterococci co-colonization among nursing facility patients. Am. J. Infect. Control.

[14] Hasan N., Cao J., Lee J., Hlaing S.P., Oshi M.A., Naeem M., Ki M.-H., Lee B.L., Jung Y., Yoo J.-W. (2019). Bacteria-Targeted Clindamycin Loaded Polymeric Nanoparticles: Effect of Surface Charge on Nanoparticle Adhesion to MRSA, Antibacterial Activity, and Wound Healing. Pharmaceutics.

[15] Bogdan C. (2001). Nitric oxide and the immune response. Nat. Immunol..

[16] Schäffer M.R., Tantry U., Gross S.S., Wasserkrug H.L., Barbul A. (1996). Nitric oxide regulates wound healing. J. Surg. Res..

[17] Wallace J.L. (2005). Nitric oxide as a regulator of inflammatory processes. Mem. Inst. Oswaldo Cruz.

[18] Smith A.W. (2005). Biofilms and antibiotic therapy: Is there a role for combating bacterial resistance by the use of novel drug delivery systems?. Adv. Drug Deliv. Rev..

[19] Malone-Povolny M.J., Maloney S.E., Schoenfisch M.H. (2019). Nitric Oxide Therapy for Diabetic Wound Healing. Adv. Healthc. Mater..

[20] Hasan N., Cao J., Lee J., Naeem M., Hlaing S.P., Kim J., Jung Y., Lee B.-L., Yoo J.-W. (2019). PEI/NONOates-doped PLGA nanoparticles for eradicating methicillin-resistant Staphylococcus aureus biofilm in diabetic wounds via binding to the biofilm matrix. Mater. Sci. Eng. C.

[21] Jones M.L., Ganopolsky J.G., Labbé A., Wahl C., Prakash S. (2010). Antimicrobial properties of nitric oxide and its application in antimicrobial formulations and medical devices. Appl. Microbiol. Biotechnol..

[22] Carpenter A.W., Schoenfisch M.H. (2012). Nitric oxide release: Part II. Therapeutic applications. Chem. Soc. Rev..

[23] Schairer D.O., Chouake J.S., Nosanchuk J.D., Friedman A.J. (2012). The potential of nitric oxide releasing therapies as antimicrobial agents. Virulence.

[24] Privett B.J., Broadnax A.D., Bauman S.J., Riccio D.A., Schoenfisch M.H. (2012). Examination of bacterial resistance to exogenous nitric oxide. Nitric Oxide.

[25] Le Q.-V., Choi J., Oh Y.-K. (2018). Nano delivery systems and cancer immunotherapy. J. Pharm. Investig..

[26] Zeb A., Arif S.T., Malik M., Shah F.A., Din F.U., Qureshi O.S., Lee E.-S., Lee G.-Y., Kim J.-K. (2018). Potential of nanoparticulate carriers for improved drug delivery via skin. J. Pharm. Investig..

[27] Nurhasni H., Cao J., Choi M., Kim I., Lee B.L., Jung Y., Yoo J.-W. (2015). Nitric oxide-releasing poly (lactic-co-glycolic acid)-polyethylenimine nanoparticles for prolonged nitric oxide release, antibacterial efficacy, and in vivo wound healing activity. Int. J. Nanomed..

[28] Hetrick E.M., Shin J.H., Paul H.S., Schoenfisch M.H. (2009). Anti-biofilm efficacy of nitric oxide-releasing silica nanoparticles. Biomaterials.

[29] Kafshgari M.H., Cavallaro A., Delalat B., Harding F.J., McInnes S.J., Mäkilä E., Salonen J., Vasilev K., Voelcker N.H. (2014). Nitric oxide-releasing porous silicon nanoparticles. Nanoscale Res. Lett..

[30] Ma X., Cheng Y., Jian H., Feng Y., Chang Y., Zheng R., Wu X., Wang L., Li X., Zhang H. (2019). Hollow, Rough, and Nitric Oxide-Releasing Cerium Oxide Nanoparticles for Promoting Multiple Stages of Wound Healing. Adv. Healthc. Mater..

[31] Pieretti J.C., Seabra A.B. (2020). Nitric Oxide-Releasing Nanomaterials and Skin Infections. Nanotechnology in Skin, Soft Tissue, and Bone Infections.

[32] Niska K., Zielinska E., Radomski M.W., Inkielewicz-Stepniak I. (2018). Metal nanoparticles in dermatology and cosmetology: Interactions with human skin cells. Chem. Biol. Interact..

[33] Szmyd R., Goralczyk A.G., Skalniak L., Cierniak A., Lipert B., Filon F.L., Crosera M., Borowczyk J., Laczna E., Drukala J. (2013). Effect of silver nanoparticles on human primary keratinocytes. Biol. Chem..

[34] Lewinski N., Colvin V., Drezek R. (2008). Cytotoxicity of nanoparticles. Small.

[35] Broniowska K.A., Diers A.R., Hogg N. (2013). S-nitrosoglutathione. Biochim. Biophys. Acta Gen. Subj..

[36] Forman H.J. (2016). Glutathione–From antioxidant to post-translational modifier. Arch. Biochem. Biophys..

[37] Champeau M., Póvoa V., Militão L., Cabrini F.M., Picheth G.F., Meneau F., Jara C.P., de Araujo E.P., de Oliveira M.G. (2018). Supramolecular poly (acrylic acid)/F127 hydrogel with hydration-controlled nitric oxide release for enhancing wound healing. Acta Biomater..

[38] Amadeu T.P., Seabra A.B., De Oliveira M.G., Costa A.M. (2007). Venereology. S-nitrosoglutathione-containing hydrogel accelerates rat cutaneous wound repair. J. Eur. Acad. Dermatol. Venereol..

[39] Lee J., Hlaing S.P., Cao J., Hasan N., Ahn H.-J., Song K.-W., Yoo J.-W. (2019). In Situ Hydrogel-Forming/Nitric Oxide-Releasing Wound Dressing for Enhanced Antibacterial Activity and Healing in Mice with Infected Wounds. Pharmaceutics.

[40] Kim J.O., Noh J.-K., Thapa R.K., Hasan N., Choi M., Kim J.H., Lee J.-H., Ku S.K., Yoo J.-W. (2015). Nitric oxide-releasing chitosan film for enhanced antibacterial and in vivo wound-healing efficacy. Int. J. Biol. Macromol..

[41] Duong H.T., Kamarudin Z.M., Erlich R.B., Li Y., Jones M.W., Kavallaris M., Boyer C., Davis T.P. (2013). Intracellular nitric oxide delivery from stable NO-polymeric nanoparticle carriers. Chem. Commun..

[42] Marcato P.D., Adami L.F., de Melo Barbosa R., Melo P.S., Ferreira I.R., de Paula L., Duran N., Seabra A.B. (2013). Development of a sustained-release system for nitric oxide delivery using alginate/chitosan nanoparticles. Curr. Nanosci..

[43] Wu W., Gaucher C., Diab R., Fries I., Xiao Y.-L., Hu X.-M., Maincent P., Sapin-Minet A. (2015). Biopharmaceutics. Time lasting S-nitrosoglutathione polymeric nanoparticles delay cellular protein S-nitrosation. Eur J. Pharm. Biopharm..

[44] Lee H.J., Park D.J., Choi G.H., Yang D.-N., Heo J.S., Lee S.C. (2016). pH-Responsive mineralized nanoparticles as stable nanocarriers for intracellular nitric oxide delivery. Colloids Surf. B.

[45] Yoo J.-W., Acharya G., Lee C.H. (2009). In vivo evaluation of vaginal films for mucosal delivery of nitric oxide. Biomaterials.

[46] Valencia P.M., Hanewich-Hollatz M.H., Gao W., Karim F., Langer R., Karnik R., Farokhzad O.C. (2011). Effects of ligands with different water solubilities on self-assembly and properties of targeted nanoparticles. Biomaterials.

[47] Malvern Instruments (2004). Zetasizer Nano Series User Manual.

[48] Danafar H., Rostamizadeh K., Hamidi M. (2018). Polylactide/poly (ethylene glycol)/polylactide triblock copolymer micelles as carrier for delivery of hydrophilic and hydrophobic drugs: A comparison study. J. Pharm. Investig..

[49] Heggers J.P., Robson M.C., Doran E.T. (1969). Quantitative assessment of bacterial contamination of open wounds by a slide technique. Trans. R. Soc. Trop. Med. Hyg..

[50] Siepmann J., Faisant N., Benoit J.-P. (2002). A New Mathematical Model Quantifying Drug Release from Bioerodible Microparticles Using Monte Carlo Simulations. Pharm. Res..

[51] Zolnik B.S., Leary P.E., Burgess D.J. (2006). Elevated temperature accelerated release testing of PLGA microspheres. J. Control. Release.

[52] Aragón D., Rosas J., Martínez F. (2014). Effect of the ibuprofen solubility in acetone and dichloromethane on the drug release profiles from PLGA microspheres. Latin Am. Appl. Res..

[53] Carpenter A.W., Worley B.V., Slomberg D.L., Schoenfisch M.H. (2012). Dual action antimicrobials: Nitric oxide release from quaternary ammonium-functionalized silica nanoparticles. Biomacromolecules.

[54] Raghupathi K.R., Koodali R.T., Manna A.C. (2011). Size-dependent bacterial growth inhibition and mechanism of antibacterial activity of zinc oxide nanoparticles. Langmuir.

[55] Macherla C., Sanchez D.A., Ahmadi M., Vellozzi E.M., Friedman A.J., Nosanchuk J.D., Martinez L.R. (2012). Nitric oxide releasing nanoparticles for treatment of Candida albicans burn infections. Front. Microbiol..

[56] Barone M., Sciortino M.T., Zaccaria D., Mazzaglia A., Sortino S. (2008). Nitric oxide photocaging platinum nanoparticles with anticancer potential. J. Mater. Chem..

